# AIDS Clinical Research in Spain—Large HIV Population, Geniality of Doctors, and Missing Opportunities

**DOI:** 10.3390/v10060293

**Published:** 2018-05-30

**Authors:** Vicente Soriano, José M. Ramos, Pablo Barreiro, Jose V. Fernandez-Montero

**Affiliations:** 1 Infectious Diseases Unit, La Paz University Hospital, 28046 Madrid, Spain; pm.barreiro@gmail.com; 2 UNIR Health Sciences School, 28040 Madrid, Spain; 3 Department of Internal Medicine, General University Hospital, 03010 Alicante, Spain; jramosrincon@yahoo.es; 4 Department of Infectious Diseases, University Hospital Crosshouse, Kilmarnock KA2 0BE, UK; jvicfer@gmail.com

**Keywords:** HIV research, HIV prevention, HIV education, antiretroviral therapy, Spain, hepatitis, AIDS history

## Abstract

The first cases of AIDS in Spain were reported in 1982. Since then over 85,000 persons with AIDS have been cumulated, with 60,000 deaths. Current estimates for people living with HIV are of 145,000, of whom 20% are unaware of it. This explains the still high rate of late HIV presenters. Although the HIV epidemic in Spain was originally driven mostly by injection drug users, since the year 2000 men having sex with men (MSM) account for most new incident HIV cases. Currently, MSM represent over 80% of new yearly HIV diagnoses. In the 80s, a subset of young doctors and nurses working at Internal Medicine hospital wards became deeply engaged in attending HIV-infected persons. Before the introduction of antiretrovirals in the earlier 1990s, diagnosis and treatment of opportunistic infections was their major task. A new wave of infectious diseases specialists was born. Following the wide introduction of triple combination therapy in the late 1990s, drug side effects and antiretroviral resistance led to built a core of highly devoted HIV specialists across the country. Since then, HIV medicine has improved and currently is largely conducted by multidisciplinary teams of health care providers working at hospital-based outclinics, where HIV-positive persons are generally seen every six months. Antiretroviral therapy is currently prescribed to roughly 75,000 persons, almost all attended at clinics belonging to the government health public system. Overall, the impact of HIV/AIDS publications by Spanish teams is the third most important in Europe. HIV research in Spain has classically been funded mostly by national and European public agencies along with pharma companies. Chronologically, some of the major contributions of Spanish HIV research are being in the field of tuberculosis, toxoplasmosis, leishmaniasis, HIV variants including HIV-2, drug resistance, pharmacology, antiretroviral drug-related toxicities, coinfection with viral hepatitis, design and participation in clinical trials with antiretrovirals, immunopathogenesis, ageing, and vaccine development.

## 1. Introduction

The first cases of AIDS in Spain were identified in 1982 [1,2]. They corresponded to hemophiliacs and homosexual males. Since then, over 85,000 persons with AIDS have been cumulated (male 70%), with 60,000 deaths. However, estimates for people living with HIV in Spain are roughly of 145,000, of whom 20% are unaware of it. This fact largely explains the high rate of late HIV presenters [3]. Although the HIV epidemic in Spain was originally driven mostly by injection drug users [4], since the year 2000 it steadily switched to men having sex with men (MSM). Currently, MSM represent over 80% of new HIV diagnoses, nearly 4000 yearly.

In the 80s, a group of doctors and nurses working at Internal Medicine hospital wards became engaged in attending HIV-infected persons. Before the introduction of antiretrovirals in the 1990s, diagnosis and treatment of opportunistic infections was their major task. A new wave of infectious diseases specialists was born. Following the wide introduction of triple combination therapy in the late 1990s, development of drug side effects and resistance led to built a core of highly devoted HIV specialists across the country. Today’s HIV medicine in Spain is largely represented by doctors and nurses working at hospital-based outclinics, where HIV persons are generally seen every six months. Antiretroviral therapy is currently prescribed to roughly 75,000 persons, almost all attended at clinics belonging to the government health public system.

**Figure 1:**
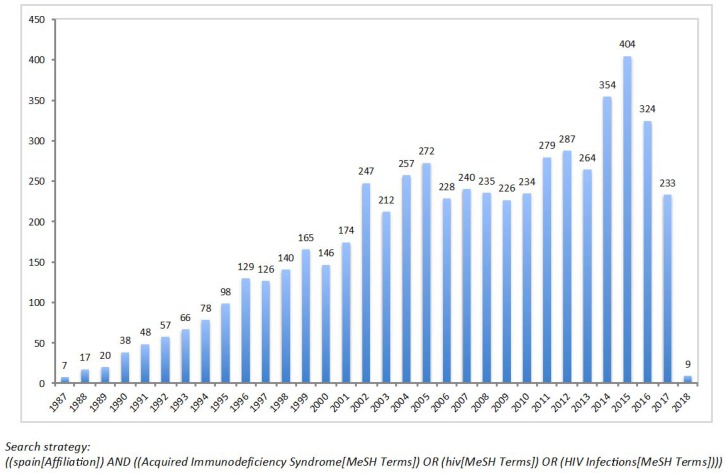
Number of publications on HIV/AIDS recorded yearly in PubMed by Spanish authors.

HIV research in Spain has classically been funded mostly by national and European public agencies along with pharma companies, but the contribution of the latest has significantly declined largely due to increased regulatory issues. Overall the impact of publications by Spanish teams working on HIV is within the three most important in Europe, according to the Science citation index [5] or PubMed. Figure 1 records the yearly number of publications by Spanish authors. Three international journals have recorded the major contribution of Spaniards in the HIV field, namely *AIDS*, *Clinical Infectious Diseases*, and *Journal of Antimicrobial Chemotherapy* [5]. Three Spanish groups, namely IrsiCaixa and Hospital Clinic in Barcelona, and Hospital Carlos III in Madrid have been within the 25 top list of HIV research teams with the greatest scientific impact worldwide (www.aidshivresearch.com).

Chronologically, some of the major contributions of Spanish HIV research are recorded in Table 1. In this overview we will address separately each of these topics, highlighting distinct studies that we consider as the most relevant. We want to apologize in advance for any missing publication that might fit as well in this review and unfortunately is not recorded due to space constraints.

## 2. Tuberculosis and HIV

The HIV epidemic in Spain during the 1980s and 1990s was largely dominated by heroin injection drug users at the largest urban areas [4]. Poor socioeconomic conditions of this group contributed to explain the rebound in tuberculosis seen in Spain at that time. Atypical forms of the disease, including meningitis [6] and splenic abscesses [7] were reported.

Beginning in the 1990s, outbreaks of multidrug-resistant tuberculosis (MDR-TB) were reported in hospitals and prisons in the eastern United States. From 1991 to 1995, MDR-TB was diagnosed in 47 HIV-infected patients and one medical doctor at the HIV ward at Hospital Carlos III in Madrid. Unfortunately, all but one died. The outbreak was produced by nosocomial transmission of *Mycobacterium bovis* [8], from were inadvertently a few patients transferred the disease to another Madrid clinics [9].

After year 2000, improvement in socioeconomic conditions, drastic declines in injection drug use, and the success of antiretroviral therapy led to an extraordinary drop of tuberculosis in the HIV population in Spain. Currently, HIV-associated tuberculosis is mainly diagnosed in immigrants (Africans, Latin Americans, and Eastern Europeans) and rarely among native Spaniards [10].

## 3. Toxoplasmosis and Leishmaniasis

During the first years of the AIDS epidemic, *Pneumocystis jiroveci* pneumonia was the most frequent classical opportunistic infection at first presentation of HIV-positive individuals in Spain. However, geographical, socioeconomic, and lifestyle unique features explained that a few conditions were overrepresented with respect to other countries. This is the case for toxoplasmosis and leishmaniasis that consequently attracted major attention.

Toxoplasmosis: Neurological disease due to *Toxoplasma gondii* typically developed in HIV-infected persons with very low CD4 counts that were not taken cothrimoxazole as prophylaxis for Pneumocystis [11]. Multiple bilateral cerebral abscesses with inflammatory component and focal neurological deficits were the most common clinical presentation. Antibiotic treatment was successful in two-thirds of acute events, but relapses were common thereafter. Spanish authors pioneered several studies on secondary prophylaxis to prevent toxoplasmosis recurrences in this population [12]. After the advent of triple antiretroviral therapy, Spanish groups were within the first to prove that immune recovery could restore immune responses [13], allowing discontinuation of toxoplasma prophylaxis [14,15].

Leishmaniasis: In the Mediterranean basin, *Leishmania infantum* was a major opportunistic parasite in persons with HIV and low CD4 counts. Roughly 5–10% of AIDS patients in Spain suffered from visceral leishmaniasis before the advent of triple antiretroviral therapy [16]. In this population, leishmania behaved as an opportunistic infection [17], with atypical clinical manifestations [18] and large parasitic amounts that even allowed visualization of parasitic forms in peripheral blood smears [19]. Relapses were frequent following treatment of acute symptomatic episodes of kala-azar, and secondary prophylaxis was needed. Spanish teams were pioneers assessing schedules and dosing pentavalent antimonials and thereafter liposomal amphotericin-B for treating visceral leishmaniasis in HIV-infected patients [20,21,22].

## 4. HIV-1 Variants and Spanish HIV-2 Network

Immigration from developing regions has resulted in an increased rate of non-B subtypes in the HIV population in Spain. These HIV-1 non-B variants have spread among native Spaniards. Since the year 2000, non-B subtypes represent over 20% of new HIV diagnoses in Spain, with CRF02_AG, G, A, and C being the most common. In native Spaniards, the current rate is above 10% [23]. Although natural genetic variability at the pol gene might account for differences in antiretroviral drug susceptibility and selection of resistance patterns across HIV-1 clades, a large study conducted in Madrid concluded that this is not the case [24].

HIV-1 group O: The first cases of HIV-1 group O infection in Spain were identified in 1996 in on heterosexual couple, both native Spaniards. The husband, however, had been working in Equatorial Guinea, a former Spanish colony in West Africa, were he admitted having had several sex partners [25]. To date, less than 10 cases of HIV-1 group O have been reported in Spain. Management of these individuals was difficult because viral load testing was not reliable at that time and most non-nucleoside reverse transcriptase inhibitors are not active [26]. Fortunately, protease inhibitors, integrase inhibitors, and even etravirine exert antiviral activity against group O [27].

HIV-2: It is a neglected virus despite estimates of 1–2 million people infected worldwide. HIV-2 is less efficiently transmitted than HIV-1 by sex. Although AIDS may develop in HIV-2 carriers, it takes on average 25–30 years. In contrast with HIV-1 infection, there is no global pandemic caused by HIV-2, remaining the virus largely confined to West Africa. Globally, HIV-2 infections are steadily declining over time [28].

A national registry of HIV-2 cases exists in Spain since 1988, when the first individuals with HIV-2 infection were identified. They were three male of West African origin living in Barcelona [29]. Since then, a total of 338 cases have been reported at the Spanish HIV-2 registry. Roughly one-third presented with CD4 counts <200 cells/μL and/or AIDS clinical events. Plasma HIV-2 RNA was undetectable at baseline in 40% [30]. To date, one-third of HIV-2 carriers have received antiretroviral therapy, being integrase inhibitors used by 32 individuals [30,31].

Overall, 72% of HIV-2 carriers in Spain are Sub-Saharan Africans. Although most cases are living around the largest urban areas (Barcelona and Madrid), two further foci of HIV-2 have been found in the north-northwest and in the southeast coasts of Spain. In the northern border, predominantly native Spaniard seamen that had worked in the West African coasts. In 2005, a cluster of homosexual men with HIV-2 was identified in the Basque country [32]. The concentration of cases in the southeast and the Canary islands is mostly represented by illegal immigrants recently arrived from West Africa [30]. Coinfection of HIV-1 and HIV-2 was found in 9% of the whole HIV-2 cohort. Missing dual infections can be harmful [33]. Based on distinct serosurveys, roughly 5000 persons are currently living with HIV-2 in Spain [30].

## 5. HIV Drug Resistance Platform

The first years of antiretroviral therapy were characterized by mono or dual therapy and treatment of patients with low CD4 counts. The prescription of suboptimal therapy in patients with high viral load uniformly led to selection of drug resistance. The benefit of initial antiretroviral regimens was only transient and, therefore, plans for rescue were considered in advance. In this scenario, resistance and cross-resistance became a crucial aspect of drug development and treatment-decision making. Most large Spanish teams developed laboratory facilities for clinical virology that complemented their clinical tasks. The integration of clinicians, virologists, and immunologists in specialized units was a critical factor for subsequent achievements of HIV research in Spain.

Another major step was the decision by the Spanish government to fund a national AIDS research network (RIS, Red de Investigación en SIDA). One of the platforms was devoted to antiretroviral resistance. This group produced important publications in the field [34,35,36,37,38]. Moreover, a national database with individual records from Spanish clinics was built along with a free website for interpretation of HIV drug resistance mutations [39].

Transmission of HIV harboring drug resistance was a major issue during the 1990s and 2000s [40]. Baseline resistance testing became recommended to all newly diagnosed HIV individuals in order to assist selection of the most convenient initial treatment. Subsequently, with the advent and widespread use of newer, more potent, well tolerated, and convenient antiretrovirals, drug resistance has steadily become less relevant. There are currently good options for treatment failures and transmission of drug-resistant viruses has significantly declined [41,42]. Today’s interest for HIV drug resistance testing has vanished in clinics and the field is no longer a top area of research.

## 6. HIV Pharmacokinetics and Pharmacogenetics

The first wave of antiretrovirals was often associated with narrow therapeutic windows. Consequently, suboptimal antiviral exposure or conversely side effects associated with overexposure were a major concern. Spanish researchers were pioneers in examining the clinical relevance of plasma concentrations of drugs, such as efavirenz or tenofovir, on neuropsychiatric effects and kidney abnormalities, respectively [43,44].

More recently, ageing of the HIV population, along with added co-morbidities that require specific medications, has focused the attention on antiretroviral drug interactions. Adequate knowledge may permit maximizing antiviral efficacy and avoid drug-related toxicities.

The most frequent drug interactions modify drug metabolism by inducing or inhibiting the cytochrome P450, leading to abnormal drug exposures. Through this mechanism, HIV protease inhibitors, especially when co-formulated with ritonavir or cobicistat as pharmacoenhancers, and non-nucleoside reverse transcriptase inhibitors interact with other medications. In contrast, nucleoside analogues, which do not or only marginally affect CYP450, are relatively free of significant pharmacokinetic interactions. However, exposure to nucleos(t)ide analogs may be influenced by induction/inhibition of drug transporters (i.e., P-glycoprotein) as well as by pharmacodynamic interference with other antivirals or cancer drugs.

Fortunately, most integrase inhibitors do not exhibit significant drug interactions. Advances in genomic techniques have facilitated the introduction of pharmacogenetics in HIV medicine. Although the best example is HLA-B5701 typing to prevent abacavir-associated hypersensitivity, Spanish authors have contributed significantly to unveil many other aspects of antiretroviral pharmacogenetics [45,46,47].

## 7. Lipodystrophy, Metabolic Abnormalities, and Cardiovascular Risk

More than 20 years after the introduction of triple combinations, HIV/HAART-associated lipodystrophy syndrome (HALS) still shadows the huge success of antiretroviral therapy [48,49]. However, substantial progress has been made in understanding pathogenic mechanisms such as host genetic determinants, the impact of HIV infection per se, and the role of antiretroviral therapy. Spanish researchers contributed significantly to characterizing the role of distinct drugs and ways to prevent it [50,51].

Pharmacological interventions to treat this condition have yielded mostly disappointing results, and the only intervention that offers an immediate esthetical improvement is plastic surgery. In the context of long-term antiretroviral therapy, fat toxicity still would remain a concern [52].

Following the replacement of the first wave of antiretrovirals associated with lipodystrophy (stavudine, zidovudine, didanosine, etc.), lipid abnormalities and increased cardiovascular risk emerged as a serious threat for antiretroviral-treated patients [53]. Spanish teams were pioneers in conducting studies to prevent and manage these complications. Switch studies, such as NEFA [54] and other smaller trials [55,56,57,58], represented major steps in the field.8. Viral Hepatitis B and C Coinfection

**Figure 2:**
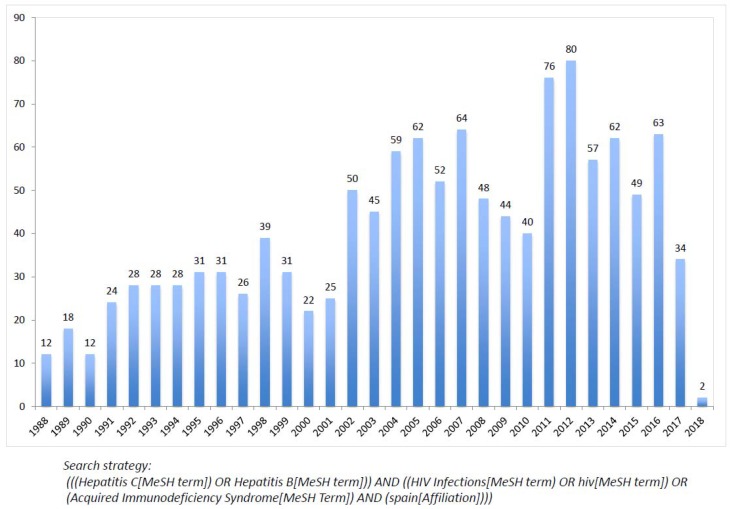
Number of publications recorded yearly in PubMed on HIV and hepatitis B/C coinfection by Spanish authors.

Given that injection drug users dominated the HIV epidemic in Spain during the first two decades [4], the high rate of viral hepatitis B, C, and delta in this group requested and attracted much attention [59,60,61]. This is contrast with conditions such as Kaposi’s sarcoma, linked to gay men, that are less common and that demanded less attention. Figure 2 records the yearly number of publications recorded in PubMed on HIV-hepatitis coinfection by Spanish authors.

From the beginning of the AIDS epidemic, it became clear that chronic viral liver disease behaved worst in the HIV population [59,62]. However, treatment options for viral hepatitis were very poor until 2010, and mostly relied on interferon alpha [60,61]. Precisely, one of the major trials with interferon monotherapy in HIV–HCV coinfected patients was run in Spain in the mid-1990s [63]. With the addition of ribavirin and the advent of weekly subcutaneous pegylated interferon, new studies attempted distinct strategies to improve HCV cure rates in HIV-coinfected patients. National multicenter studies using schedules similar to those conducted in monoinfected patients [64] or using innovative strategies, such as in PRESCO [65] and PERICO [66], were pioneered in the field.

For many years, Spanish teams led European epidemiological studies carried out by EuroSIDA on viral hepatitis B [67], C [68], and delta [69]. Likewise, Spaniards coordinated worldwide initiatives to confront the impact of chronic hepatitis B [70] and C [71], including the hepatotoxicity of antiretroviral agents that was enhanced in HIV–HCV coinfected patients [72,73]. A major achievement was the demonstration of the influence of interferon lambda (*IL28B*) gene polymorphisms as predictors of interferon response [74] and liver disease progression in HIV–HCV coinfection [75]. In parallel, Spanish groups were leaders demonstrating that liver fibrosis staging could be adequately assessed in coinfected patients using elastometry, an easy non-invasive tool, instead of a liver biopsy [76]. Subsequently, an algorithm (named *Prometheus*) [77] based on baseline variables was developed and posted freely available in the website that allowed to predict treatment outcomes using peginterferon-ribavirin, and in this way assist treatment decision making. The algorithm was endorsed by the European AIDS Clinical Society (EACS).

**Figure 3:**
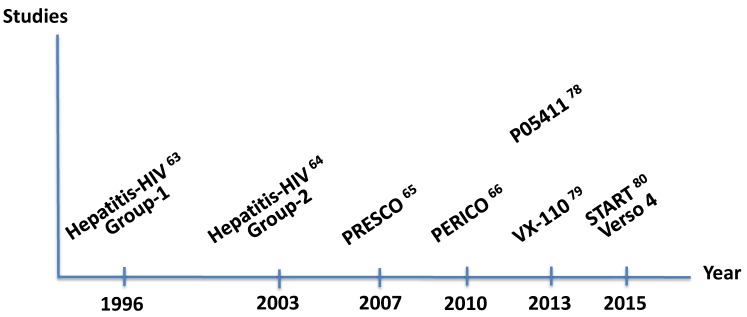
Major prospective clinical trials on treatment of hepatitis C in HIV coinfection led by Spanish researchers.

The participation of Spanish teams in registrational trials with new oral HCV antivirals in HIV–HCV coinfected patients was important, especially testing the first wave of drugs, such as boceprevir [78], telaprevir [79], or faldaprevir [80]. Figure 3 records the most cited studies led or co-led by Spanish investigators on hepatitis C therapy in HIV-coinfected patients. The advent of all-oral direct-acting antivirals for treating hepatitis C [81] and the wide use of tenofovir as treatment for chronic hepatitis B [82] have dramatically changed outcomes of viral hepatitis in HIV patients. At this time, only hepatitis delta remains as a major threat [83,84]. On the horizon, however, fatty liver disease is emerging as a new challenge in HIV patients [59,85], given the high rate of metabolic abnormalities in this population, besides more frequent alcohol abuse. During the last couple of years, a team from Seville is leading in this new field [85].

Spain is one of the world leading countries performing solid organ transplantation. Not surprisingly, it has pioneered conducting transplants in HIV-positive patients, including hepatic allografts. A large national cohort of over 300 liver transplants in HIV+ recipients, mostly with HCV coinfection was established a decade ago [86]. Although survival rates are slightly lower than in HIV-negative counterparts, they are over 55% at five years [86]. Interestingly, survival is not worsened by HIV in liver transplant recipients with hepatocellular carcinoma, being above 65% at five years [87]. Moreover, incident liver cancer in allografts is not more frequent in the HIV setting [88]. Outcomes in this population are rapidly improving following the advent of new direct acting antivirals for hepatitis C [89].

## 8. Non-Cirrhotic Portal Hypertension in HIV

In year 2006, close attention to a subset of HIV-infected individuals on long-term antiretroviral therapy and well controlled HIV infection that presented with liver decompensation events in the absence of any known hepatic injury, including alcohol abuse, viral hepatitis, etc. indicted a new life-threatening syndrome [90]. Interestingly, whereas severe portal hypertension was a major feature, advanced liver fibrosis was not. Exposure to didanosine was unveiled as the cause of this cryptogenic liver disease [90,91]. Liver biopsy depicted unique features of obliteration of small portal veins as the most distinctive histological finding [92].

Interestingly, this condition may develop in HIV patients superpose to other more common liver conditions (i.e., chronic hepatitis C), producing disproportionate portal hypertension and complicating diagnosis, management, and prognosis [93]. As part of a collaborative European project, a host genetic predisposition for didanosine hepatopathy was more recently characterized [94].

## 9. Reproductive Options in HIV

Heterosexual contact is the major route of HIV transmission worldwide. Fortunately, the achievement of undetectable viremia with antiretroviral therapy is one of the best ways to halt sexual HIV transmission. This observation has represented a major benefit for HIV-serodiscordant couples, opening the door for having children and family to HIV+ persons [95,96,97]. Personally (V.S.), it always come to my mind the discussions I had on this issue with some of our first HIV-infected hemophiliacs, as well as with one HIV-positive young lady that now is a proud mother of five healthy children.

At community level, the proportion of HIV-infected individuals on antiretroviral therapy with undetectable viremia has been inversely correlated with the rate of new HIV infections, especially in places with large communities of sexually active homosexual men [98]. More recently, the benefit of antiretroviral therapy for halting HIV transmission has been extended for its use as prevention, mostly as pre-exposure prophylaxis (PrEP) [99]. Whereas Truvada^®^ taken either daily, intermittently, or at demand reduces the risk of HIV acquisition in persons engaged in high-risk sexual practices, other sexually transmitted infections are on the rise, including gonorrhea, syphilis, and even acute hepatitis C [100,101,102]. This observation reinforces that education and behavioral interventions should complete drug administration to really make a benefit [103,104]. Recently, concerns rose on the risk of transmission and/or acquisition of drug-resistant HIV in the PrEP context [105].

## 10. Antiretroviral Clinical Trials

Most phase two to four clinical trials funded by the international pharma industry have included Spanish teams when involving Europe. In some of these international studies, Spaniards have been leaders or co-leaders. On the other hand, many investigator-driven prospective studies on antiretroviral therapy have been developed by Spanish researchers. In particular, Spaniards have been pioneers in the design of simplification (or switch) studies, generally carried out in HIV patients with suppressed HIV replication under distinct regimens and in order to improve convenience and reduce side effects without compromising viral control.

**Figure 4:**
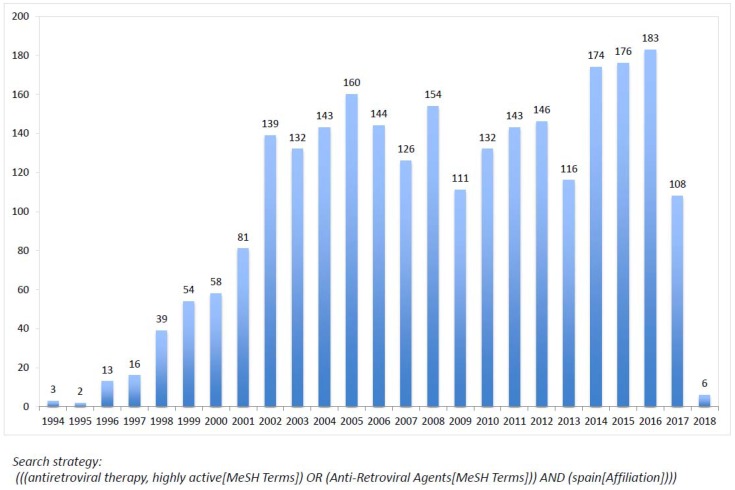
Number of publications recorded yearly in PubMed on antiretroviral therapy by Spanish authors.

Given the relatively large size of the HIV population in Spain and the almost universal access to antiretroviral therapy, Spanish specialists have contributed substantially to recording experience in real-life antiretroviral therapy, including both safety and efficacy. Figure 4 records the yearly number of publications by Spanish teams recorded in PubMed.

**Figure 5:**
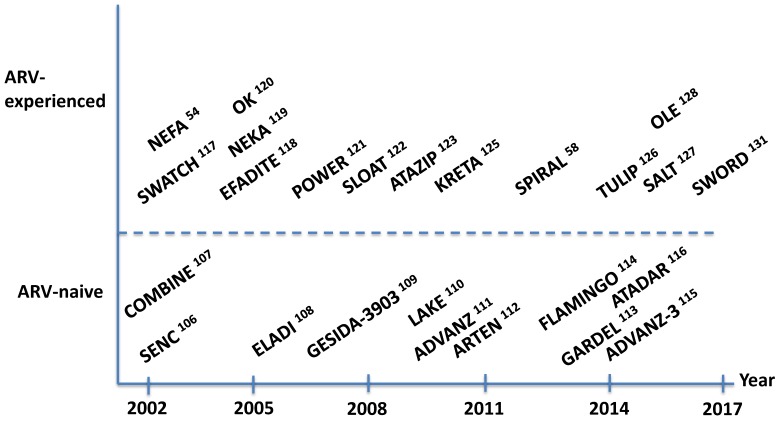
Major prospective clinical trials on antiretroviral therapy in adults led by Spanish researchers.

Figure 5 records the most cited prospective trials on antiretroviral therapy led by Spanish investigators, splitting out studies in drug-naïve [106,107,108,109,110,111,112,113,114,115,116] and treatment-experienced patients [54,58,117,118,119,120,121,122,123,124,125,126,127,128,129,130,131], the latest either as switch or rescue interventions. It must be noted, however, that experimental monotherapies or dual therapies generally with boosted protease inhibitors with or without lamivudine (i.e., less costly, easier dosing, and lower side effects) have been subject to criticism in the modern era, when several single tablet triple regimens, with more potent and well-tolerated drugs are available, and major efforts are devoted to the achievement of HIV eradication.

## 11. HIV Immunopathogenesis and Ageing

It could be thought that basic science was beyond the scope of Spanish HIV research, with clinical and therapeutic investigations being more feasible, running in parallel with the large number of infected persons. However, among others, Spaniards have contributed significantly in understanding the genetic determinants of HIV acquisition [132] and disease progression in studies conducted in HIV long-term non-progressors and elite controllers [133,134,135,136]. Likewise, they have contributed significantly in the fields of treatment intensification [137], immune recovery [138], and pathogenesis of chronic immune activation and systemic inflammation [139]. Some of these studies have been facilitated by the establishment of a national repository of human specimens [140].

The broader use of antiretroviral therapy, given to everyone and as soon as possible has lead to halting disease progression in most infected persons. HIV is currently a chronic condition and survival of carriers is nearly the same of uninfected persons. Spanish teams have been active in elucidating the mechanisms and clinical consequences of ageing in this population [54].

## 12. HIV Vaccine Development

Despite the enormous recent advances in the HIV prevention landscape, an effective vaccine remains the most promising tool to end the HIV-1 pandemic. Early enthusiasm unabated, the prospects for finding a protective HIV vaccine have been tempered after disappointing results of first trials one decade ago. One of the major obstacles in vaccine trials has been the lack of identification of good correlates of protection. Although cellular immunity, both innate and adaptive, plays a major role in HIV control, the ability to induce broadly neutralizing antibody responses is likely essential for development of a globally effective HIV vaccine. Unfortunately, human vaccine trials conducted to date have failed to elicit broad plasma neutralization of primary virus isolates [141]. However, lessons from the RV144 trial have re-energized the field. In the meantime, therapeutic vaccines that pursue enhancing immune responses and controlling disease progression in people already infected are the focus of major attention. Several Spanish teams are running up front in this path using distinct approaches, including dendritic cells [142] or modified viral vectors [143,144,145].

## 13. Other Spanish Contributions to HIV Research

Being aware that discussing 35 years of scientific contributions in HIV/AIDS by Spanish authors is something too pretentious, we would like to complete the list of major topics acknowledging that international collaborations and educational activities have been an invaluable research contribution. Both patients and doctors from Spain have contributed considerable support to national (VACH, CoRIS, etc.), European (EuroSIDA, CHAIN, CASCADE, PENTA, NEAT, SPREAD, etc.), and international (COHERE, SMART, HIDN, etc.) networks that have provided crucial information in the HIV/AIDS field.

For many years, postgraduate courses and masters, mostly run in Madrid and Barcelona, have been the major source of acquiring expertise in HIV medicine. Health care professionals coming from distinct Spanish cities and another countries, mostly from Latin America, have benefitted from these initiatives. Textbooks in Spanish with over five re-editions have been printed periodically by Spanish experts [146,147], and have contributed significantly to improve HIV care locally and overseas, especially in Latin America.

## 14. Current Spanish HIV Research

During 35 years, HIV medicine has evolved from birth to maturity with unprecedented speed. Since the early 80s, HIV care in Western countries has shifted from confronting opportunistic acute infectious processes to demand attention for chronic conditions of an ageing HIV-infected population [54,148]. Once current antiretroviral therapy has led most patients to achieve sustained and complete suppression of virus replication with few side effects, HIV care has entered a new era. Sophisticated specialized knowledge is no longer needed for providing enough good care to the vast majority of HIV persons.

Over time, most long-term complications of HIV infection will overlap with those of the general population as it ages, acknowledging that risks may be enhanced in HIV persons and therefore may appear at a younger age. Residual persistent immune activation and systemic inflammation, even under effective antiretroviral therapy, seems to largely explain this premature frailty [149]. Indeed, adequate HIV care should include prevention and treatment of cardiovascular diseases, non-AIDS cancers, renal insufficiency, osteoporosis, diabetes, neurocognitive disorders, liver diseases, and lung conditions. In Western countries, these non-communicable diseases have already become the most important source of morbidity and mortality in HIV persons [54]. Clinical research in Spain on HIV is already addressing these topics and joining international efforts to face these new issues.

The prospects for HIV eradication are currently the major focus of attention for AIDS scientists. Several Spanish teams are involved in this fascinating adventure [150,151]. At the same time, real-life experience highlights that translational medicine is crucial. No major research advance in HIV would have an impact without considering how to best reach the target populations. As an example, the lack of commitment to make accessible HIV testing and denying antiretroviral therapy to some marginal groups must be solved [152].

## 15. Future Challenges for Spanish HIV Research

Enthusiasm unabated, Spanish HIV/AIDS scientists are subject to new and unique challenges in the near future. Of note, the peak of interest about HIV/AIDS is rapidly declining as survival of patients improves and the epidemics slow down. As a result, funding is moving off the field, despite the burden of HIV being high and expected to remain disproportionately elevated in Spain.

**Figure 6:**
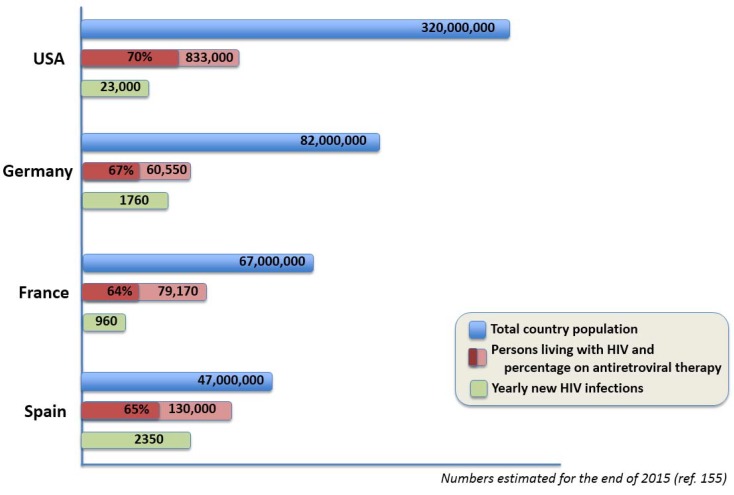
Burden of HIV infection in Spain. Comparison with other Western countries.

In particular, there is a need to revisit preventive strategies. It is remarkable that HIV incidence in Spain is still one of the largest in Western Europe [153]. The number of new HIV diagnoses per year is roughly 2.5-fold compared to France or 1.5-fold compared to Germany, despite having larger populations and similar rates of antiretroviral coverage for their HIV patients (Figure 6). Given that more than 80% of new HIV infections in Spain are occurring in MSM, mostly young native Spaniards, re-thinking education and behavioral interventions seems mandatory [3].

Ultimately, the risk of HIV acquisition depends on the amount and quality of exposure to the infected source. In other words, reducing the number of sex partners and the frequency of unprotected high-risk sex encounters (i.e., anal intercourse) should drop new infections [154,155].

As individual rights to choose sex behavior are beyond control, we must all agree that the limit is the potential harm for others [154]. In this regard, it is impressive how much effort is given to discouraging tobacco and, conversely, how little effort has been made for promoting healthy behavioral changes to confront sexually transmitted infections, including HIV. While smoking is socially poorly accepted and declining, venereal diseases are rising [100,102,103].

Another challenge for HIV/AIDS researchers is the lack of commitment and prioritization of scientific excellence. The result is that short-term vision too often leads to favoring less-qualified persons/teams. Lack of awarding the best comes along with poor fund from public agencies (government lacking conviction in science investment) as well as private groups (due to excessive legal barriers and few economic incentives).

As result of the limited research funding in Spain, there is a flux of young, promising, mature, and good researchers moving to other countries where they are given better opportunities. Efforts to keep them in Spain should be made, awarding their achievements with better salaries and social recognition. Ultimately, there is a need to build robust scientific structures and networks instead of rely on the good look of a few genial scientists. A large group of prestigious Spanish researchers has recently conveyed in a forum (*Gadea Science Foundation*) that it aims to actively advise the Spanish government on initiatives on research and science [156].

A last challenge for Spanish HIV research refers to the global degradation of the Spanish health system, with increasing excessive burocratization that mutilates autonomy, initiatives, and creativity [157]. We, Spaniards, should be proud of our achievements in HIV research despite limited funding, poor research infrastructure and difficult environment [156,157]. We should learn from what is happening in other places. An American journalist recently noted the following: “why does America pay the highest cost per person for healthcare? …the health administrative system has mushroomed into an incredibly costly bureaucratic monster that provides zero care. Add layer after layer of new complex regulations to the practice of medicine, and soon enough you need millions of paper-pushing employees to monitor compliance, enforce compliance, pursue administrative and criminal charges of non-compliance, file claims and counter-claims, defend the innocent from false accusations, write hundreds of pages of new regulations, and so on. Yes, there is a place for common-sense regulations, and procedures to vet caregivers and track standards of care, etc. However, the system is now so onerous and out of control that the practice of medicine now requires far more attorneys and compliance-regulatory-paper-pushers than it does doctors and nurses” [158]. Clearly, there is a need for re-directing efforts to what is important, the ultimate goal of the health system: the patients.

## Figures and Tables

**Table 1:** Major contributions of Spanish HIV research.

Prevention and treatment of tuberculosis in HIV Management of HIV-associated toxoplasmosis and leishmaniasis HIV variants, including HIV-2 Antiretroviral drug resistance Antiviral pharmacokinetics/pharmacogenetics Antiretroviral-associated lipodystrophy and metabolic abnormalities Management and treatment of coinfection with viral hepatitis Characterization of non-cirrhotic portal hypertension in HIV Reproductive options for HIV couples Antiretroviral clinical trials Immunopathogenesis Vaccine development
